# Dynamic transition of neuronal firing induced by abnormal astrocytic glutamate oscillation

**DOI:** 10.1038/srep32343

**Published:** 2016-08-30

**Authors:** Jiajia Li, Jun Tang, Jun Ma, Mengmeng Du, Rong Wang, Ying Wu

**Affiliations:** 1 State Key Laboratory for Strength and Vibration of Mechanical Structures, School of Aerospace, Xi’an Jiaotong University, Xi’an 710049, China; 2 College of Science, China University of Mining and Technology, Xuzhou 221116, China; 3 Department of Physics, Lanzhou University of Technology, Lanzhou 730050, China

## Abstract

The gliotransmitter glutamate released from astrocytes can modulate neuronal firing by activating neuronal N-methyl-D-aspartic acid (NMDA) receptors. This enables astrocytic glutamate(AG) to be involved in neuronal physiological and pathological functions. Based on empirical results and classical neuron-glial “tripartite synapse” model, we propose a practical model to describe extracellular AG oscillation, in which the fluctuation of AG depends on the threshold of calcium concentration, and the effect of AG degradation is considered as well. We predict the seizure-like discharges under the dysfunction of AG degradation duration. Consistent with our prediction, the suppression of AG uptake by astrocytic transporters, which operates by modulating the AG degradation process, can account for the emergence of epilepsy.

The neuronal system is composed of a large number of neurons and astrocytes, and it is confirmed that astrocytes can play important role in regulating the electric modes of activities^1^,^2^,^3^,^4^. Most of the neuronal models mainly emphasize the dynamical properties of electric activities, and often bifurcation parameters are carefully adjusted to trigger possible mode transition in electrical activities. These models are helpful to understand the synchronization problems of neurons. Gu *et al*. proposed a neuronal model to detect the possible dynamical behavior of a sciatic nerve chronic constriction injury model^5^. Multiple modes can be observed in neuronal activities, Gu *et al*. investigated the dependence of model selection on bifurcation parameter and initials selection^6^. Furthermore, Ma *et al*. proposed an improved model to describe the emergence and transition of multiple modes in electric activities by introducing magnetic flux in the original Hindmarsh-Rose neuron according to electromagnetic induction effect^7^,^8^. Some intermediate neurons are connected with autapse, a specific autapse connected to the body of neuron, which counts the emergence of intrinsic time delay in neuron^9^. The previous works confirmed that autapse connection plays important biological function by regulating the electric activities of isolate neuron and collective behaviors of neuronal network as pacemakers^10^,^11^,^12^,^13^,^14^,^15^,^16^,^17^. Particularly, coupling between neurons and astrocytes could be more reliable to understand the complex behavior of neuronal systems.

Over the past decades, our understanding of astrocytes has fundamentally changed: they were first considered as passive cells before being subsequently recognized as biologically excitable cells^1^,^2^. One form of excitability is a change in intracellular Ca^2+^ concentration, which occurs both spontaneously and in response to the neuronal activity^3^,^4^. Consequently, an elevation of Ca^2+^ concentration can induce a release of gliotransmitters from astrocytes in a Ca^2+^ -dependent manner^18^,^19^. Here, astrocytic glutamate (AG) is one of the major gliotransmitters and exerts its signal transducing effect on neurons via N-methyl-D-aspartic acid (NMDA) receptors^20^,^21^. Finally, astrocytes can “listen” and respond to neurons in a “tripartite synapse” loop (i.e., an astrocyte-neuron feedback loop)^22^,^23^.

Recent studies showed that the normal function of astrocytes is to support some physiological functions, such as neuronal synaptic information processing^24^,^25^,^26^,^27^ or synaptic plasticity^28^.

In an experimental study by Tian *et al*. in 2005^29^, the authors suggested that astrocytes may contribute to the neuronal depolarization underlying epilepsy. Also, Fellin *et al*. challenged the traditional concept that synchronous neuronal activity during seizures arises from an entirely neuronal origin, since they found that astrocytes can also induce synchronous neuronal activity^30^,^31^. Therefore, some scientists have proposed that astrocytes are likely to be potential targets for anti-epileptic therapeutic strategies^32^. Although astrocytes have been reported to play a potential role in epileptic seizure, the underlying causes are diverse and not completely understood^33^,^34^.

Computational modeling has been widely used for understanding the dynamics of neurons and neuronal networks^35^,^36^,^37^, and those dynamic characteristics of neurons predicted by modeling analysis were also proved in experimental results^38^,^39^, which verifies the significance of modeling analysis of neurons. These computational methods are also used to identify the impaired neurons underlying epilepsy^40^,^41^,^42^, and in recent years some models have been developed to study the astrocyte-induced epilepsy^43^,^44^,^45^,^46^,^47^. In the study of Nardkarni *et al*., a two-compartment neuron-astrocyte model was established to account for epilepsy in these experiments, when the astrocytic neurotransmitter receptors were over-expressed^43^,^44^. Some other neuron-astrocyte models have been developed to investigate the synchrony network epilepsy which is induced by an AG release^45^. However, few studies have paid attention to the relation between the different dynamic phases of the AG and epilepsy. Recent experiments have shown that a large amount of glutamate transporters are located in the astrocyte to uptake the AG^48^,^49^, and that a low-efficiency hydrolysis may trigger an epileptic seizure^50^. However, to the best of our knowledge, this effect has not been considered in previous modeling studies. Thus we investigated how the uptake-related AG decay process can affect the seizure dynamics.

In this paper, we incorporated the dynamics model of AG, which could well describe the decay process of AG, into a classical astrocyte-neuron feedback loop model^44^ in order to investigate how a low-efficiency AG decay affects the generation of seizure-like discharge.

With this model, we explored how an increase of AG equilibrium concentration and decay period changes the regular neuronal spiking into a seizure-like discharge. In addition, we also analyzed different phases of seizure-like discharge and the corresponding AG concentration states. Finally, we also adopted the energy cost theory of Hodgkin-Huxley model^51^ to distinguish seizure-like discharge from normal spiking.

## Model and Method

The reduced “tripartite synapse” is a three-compartment model of a somatic neuron, a dendrite and the neighboring astrocyte developed by Nardkarni and Jung^44^. In this model, a somatic neuron transfers its firing to the dendrite through electrical coupling. Subsequently, the action potential generated at the activated dendrite elicits the release of neurotransmitters that bind to the astrocyte receptors; as a consequence, the level of IP_3_ (inositol 1, 4, 5-triphosphate) in the astrocyte increases, which excites the Ca^2+^ oscillation in the astrocyte. Finally, the Ca^2+^ oscillation accelerates the glutamate increase in the extracellular space, which in turn depolarizes the somatic firing. In this paper, we focused on the dynamic model of astrocytic glutamate, which is defined in Eq. (9).

To model of the pyramidal cell and the dendrite, the well-known Pinsky-Rinzel (PR) model^52^ has been used in the classical “tripartite synapse” model. This model can well describe the main features of Na^+^ and K^+^ ion conductance of the soma and the calcium dependence of the dendrite. The action potentials of the soma (*V*_*s*_) and the dendrite (*V*_*d*_) are described by the following set of equations:


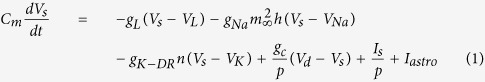



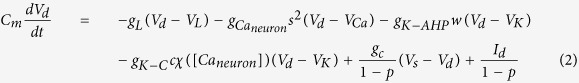


where *C*_m_ = 3.0 μFcm^−2^ represents the membrane capacitance of the soma and the dendrite. *V*_Na_ = 115.0 mV, *V*_K_ = −15.0 mV, *V*_Ca_ = 140.0 mV, *V*_L_ = 0.0 mV respectively denote the Nernst potentials of the sodium, potassium, calcium and the leakage channels. The maximal conductance of the sodium channel, the three types of potassium channels, calcium channel and the leakage channel are given as follows: g_Na_ = 30.0 mS cm^−2^, *g*_K-DR_ = 15.0 mS cm^−2^, *g*_K-AHP_ = 0.8 mS cm^−2^, *g*_K-C_ = 15.0 mS cm^−2^, g_Ca neuron_ = 10.0 mS cm^−2^ and *g*_L_ = 0.1 mS cm^−2^. g_c_ = 2.1 mS cm^−2^ represents the coupling intensity between the soma and the dentdrite, and the parameter *p* = 0.5 denotes the fraction of the cell volume taken up by the soma. For the PR model, the rest-to-spiking rheobase current for the soma and the dendrite are −0.3 μA cm^−2^ and −0.25 μA cm^−2^, *I*_s_ = 0 μA cm^−2^ representing the external stimulating current on the pyramidal soma, and *I*_d_ = 0 μA cm^−2^ representing the external stimulating current for the dendrite.

The slow inward current *I*_*astro*_ that is induced by the astrocytic glutamate shows to be proportional to the concentration of astrocytic glutamate^53^,^54^, and therefore *I*_*astro*_ gives the form:


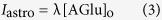


where [AGlu]_o_ denotes the extracellular AG concentration, and λ = 2.11 μA cm^−2^ μM^−1^ represents the expression level of the NMDA receptors in the soma.

The kinetic equations for the gating variables *h*, *n*, *s*, *c*, *w* give the form:


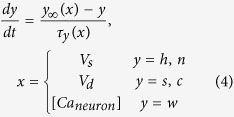


where


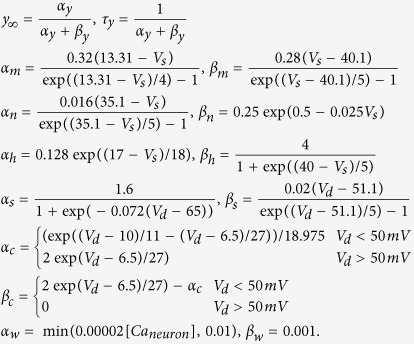


[Ca_neuron_] denotes the dimensionless free calcium concentration in the dendrite^44^


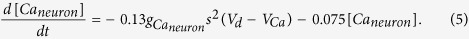


When the dendrite fires, the neurotransmitters released from the dendrite can trigger the production of IP_3_ in the neighboring astrocyte, which is modeled by Nardkarni and Jung^43^,^44^ as


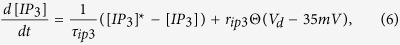


where [IP_3_]* denotes the equilibrium concentration of IP_3_, which is a secondary messenger molecule used in astrocytes to bind receptors in the endoplasmic reticulum (ER) membrane and elicit a Ca^2+^ efflux from ER. Predetermined values of [IP_3_]* = 160.0 μM and *τ*_*ip*3_ = 7 s were used according to previous experiments^55^. Subsequently, the elevation of the IP_3_ concentration induces an increase in intracellular Ca^2+^ in the astrocyte.

The Li-Rinzel model has been used to describe the calcium exchange in the astrocyte^43^,^44^,^56^, which contains three fluxes across the ER membrane: an IP_3_-dependent calcium ion channel, a pump channel, and a leaky channel. They are described as follows:


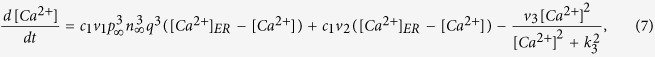



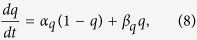


where


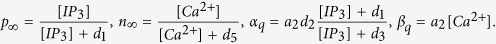


The parameters of the classical Li-Rinzel model are *c*_1_ = 0.185, *v*_1_ = 6 s^−1^, *v*_2_ = 0.11 s^−1^, *v*_3_ = 0.9 μM.s, *a*_2_ = 0.2 μM.s^−1^, *d*_1_ = 0.13 μM, *d*_2_ = 1.049 μM, *d*_3_ = 0.9434 μM, *d*_5_ = 0.08234 μM. The conservation of calcium in astrocyte implies the constraint [Ca^2+^]_ER_ = (*c*_0_-[Ca^2+^])/*c*_1_ with *c*_0_ = 2.0 μM. The parameter *q* denotes the gating variable of calcium channel in the ER membrane of the astrocyte.

The experimental evolution of AG shares similarities with astrocytic IP_3_: 1) a transient increase by the pulse of the astrocytic Ca^2+^ signal (for IP_3_, this corresponds to the voltage signal); the decaying process by the uptake of astrocytic transporters (for IP_3_, this corresponds to the IP_3_ enzyme in the astrocyte).

Regarding the dynamics aspects, we proximately use the framework of the IP_3_ model to describe the AG dynamics. Therefore, the characteristics of the AG dynamics ([AGlu]_o_) can be described as follows:


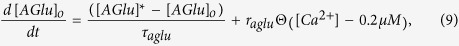


where the first term describes the degradation of AG with a degradation rate of 1/*τ*_aglu_ and an equilibrium concentration of [AGlu]*. When considering a normal uptake function of the astrocytic transporters, [AGlu]* is assumed to be approximately 0. However, when the AG uptake is suppressed, [AGlu]* would increase.

In fact, experimental results showed that astrocytes could contribute to the neuronal depolarization underlying epilepsy through an accumulation of extracellular AG^29^,^30^,^31^. Also, an abnormal AG uptake due to dysfunctional astrocytic transporters could accelerate the AG accumulation^50^. In Eq. (9), both high AG equilibrium concentration ([AGlu]*) or long degradation time constant (*τ*_aglu_) could be typical characteristics of an abnormal AG uptake state. The second term in Eq. (9) is activated when astrocytic Ca^2+^ concentration is larger than a threshold of 0.2 μM^53^,^54^ via the step function. The parameter *r*_aglu_ = 1.0 μM s^−1^ represents the quantized production of extracellular AG as [Ca^2+^] remains over 0.2 μM.

## Numerical Results and Discussion

**Figure 1:**
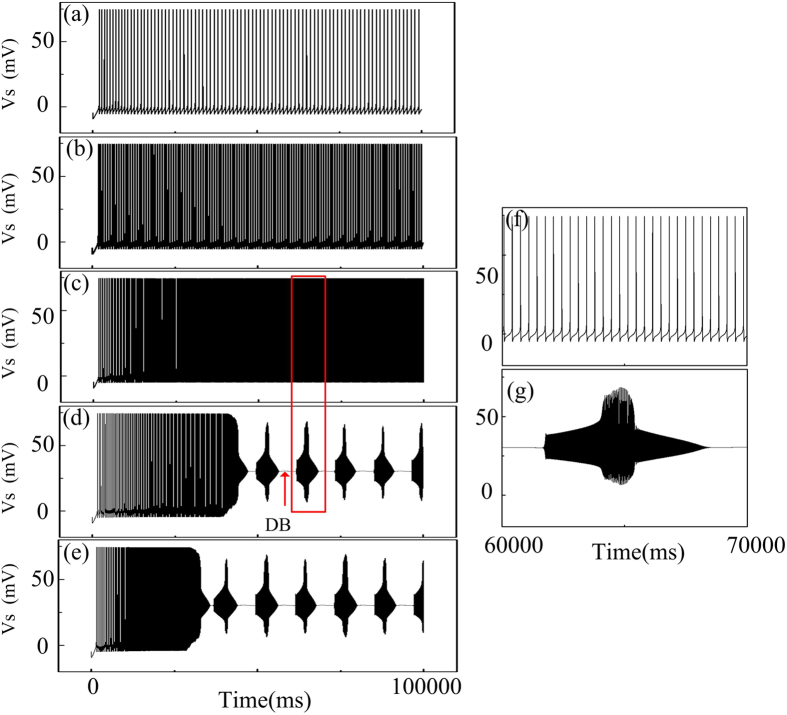
Epileptic discharge induced by the increase of [AGlu]* with *τ*_aglu_ = 10 s. The AG equilibrium concentration [AGlu]* is (**a**) 0.0 μM; (**b**) 0.1 μM; (**c**) 0.3 μM; (**d**) 0.5 μM; (**e**) 0.7 μM. Additionally, (**f**,**g**) show the neuronal discharge between 60000 ms and 70000 ms, zoomed from panels (**c**,**d**) respectively.

Figure 1 shows neuronal discharges with AG equilibrium concentration ([AGlu]*) at 0.0 μM (a), 0.1 μM (b), 0.3 μM (c), 0.5 μM (d) and 0.7 μM (e). As shown in Fig. 1(a–c), the neuronal discharge shows regular spiking when the equilibrium concentration stays lower than 0.5 μM. However, with higher level of equilibrium concentrations, such as 0.5 μM and 0.7 μM, a seizure-like discharges are present in the soma, as it can be observed in Fig. 1(d,e) respectively. Both the “depolarization block” (DB) discharge and the “refractory status epilepticus”(RSE)-like discharge are present in the time series of neuronal discharge. The DB discharge, observed in Fig. 1(d), shows a short period of hyper-resting state, and has been observed in the time series of epileptic seizure^57^,^58^,^59^. Besides, the RSE-like discharge shown in Fig. 1(f) is an enlarged version of that shown in the red box in Fig. 1(d). It can be seen in Fig. 1(g) that this discharge period shows long-period and high-rate mixed-mode oscillations (MMO). The high-rate feature can be determined from the comparison between Fig. 1(f) and Fig. 1(g). It can be seen that the neuronal firing frequency in Fig. 1(g) is much higher than the neuronal firing frequency observed in Fig. 1(f). In fact, Tian *et al*. in experiments have reported that the accumulation of AG has been a major source of neuronal epilepsy^29^,^30^,^31^, AG uptake by astrocytic transporters in some experimental results was shown to be an efficient pathway to protect neurons from epilepsies^33^,^34^. Furthermore, Hubbard *et al*.^50^ even reported various epilepsies that were induced by abnormal AG clearance process. In the present study, an elevated [AGlu]* corresponds to an abnormal AG clearance process, which could also predict the epileptic phenomena.

In order to describe the hyper-high firing rate characteristics of seizure-like discharges that differ from regular discharges, we introduced the notion of an average energy cost <H>^51^,^60^:


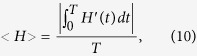



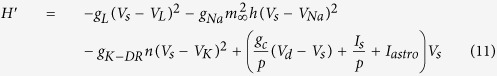


where we consider the average energy cost <H> in the time scale with T = 100000 ms. The first three terms in Eq. (11) represent the energy cost of three ion channels, the last term denoting the energy supply from the total external current, which arises from the coupling current between the soma and the dendrite. The constant stimulating current in the soma and astrocytic current is due to AG.

**Figure 2:**
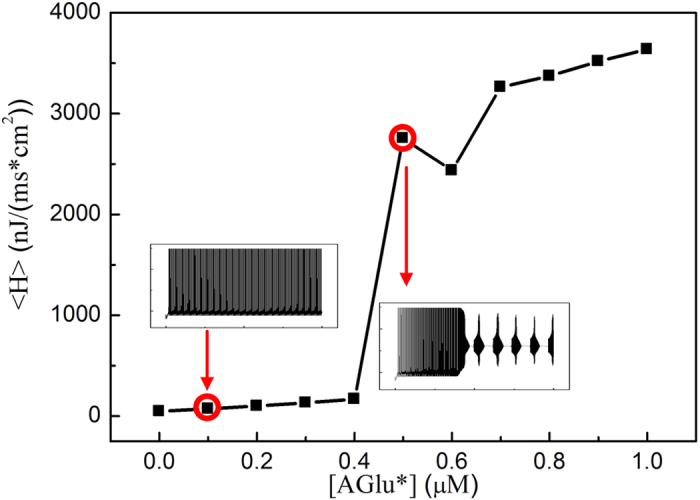
The average energy cost <H> versus the equilibrium concentration [AGlu]*. The micro-figures of neuronal discharges correspond to the cases when [AGlu]* = 0.1 μM and 0.5 μM.

Figure 2 shows the average energy cost versus the AG equilibrium concentration, It can be observed that when [AGlu]* < 0.5 μM, the average energy cost stays about 200–400 nJ/(cm^2^*ms). When [AGlu]* increases above 0.5 μM, the average energy cost shows an abrupt elevation of <H>, until the neuron start to generate seizure-like discharge. This suggests that the emergence of seizure-like discharges is accompanied by an “energy explosion”. In fact, the transition of brain electric activities corresponds to changes in multiple forms, such as characteristics of the neuronal network random matrix^61^ and presentations of the neuronal network spatiotemporal patterns^62^. In this paper, we used statistical measurement energy consumption <H> and successfully described a special transition of brain electric activities, i.e., the generation process of an epileptic seizure. In fact, experimental results have also shown that a brain seizure corresponds to a high-level energy expenditure^63^,^64^.

**Figure 3:**
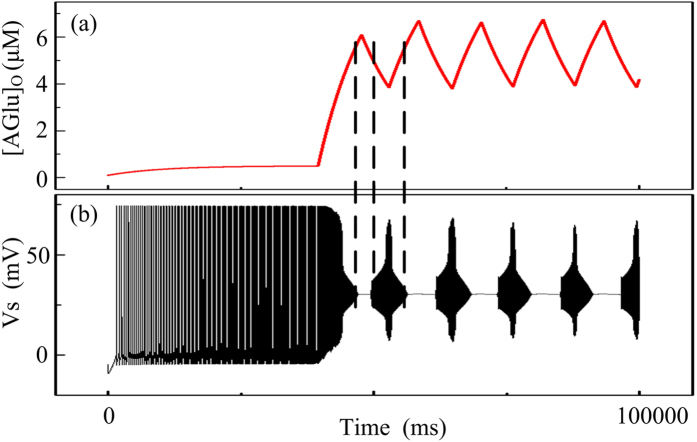
The gliotransmitter AG regulates the epileptic seizure with *τ*_aglu_ = 10 s. (**a**) AG time concentration when [AGlu]* = 0.5 μM; (**b**) the neuronal discharge when [AGlu]* = 0.5 μM.

Figure 3 shows AG oscillations and the corresponding neuronal discharges during the same time period with an equilibrium concentration of [AGlu]* = 0.5 μM. It can be seen that when the AG concentration stays at a low level, the corresponding neuronal discharges show regular spiking, However, when AG oscillations reach higher levels, the neuron starts to fire with seizure-like discharges. An interesting point is that the DB discharge is present when AG oscillations peak. Because AG offers excitable stimulus on neurons, hyper-high concentration of AG can therefore induce a neuronal electric shock. On the other hand, as AG concentration decreases from the peak, the neuronal discharge resembles RSE-like discharge.

**Figure 4:**
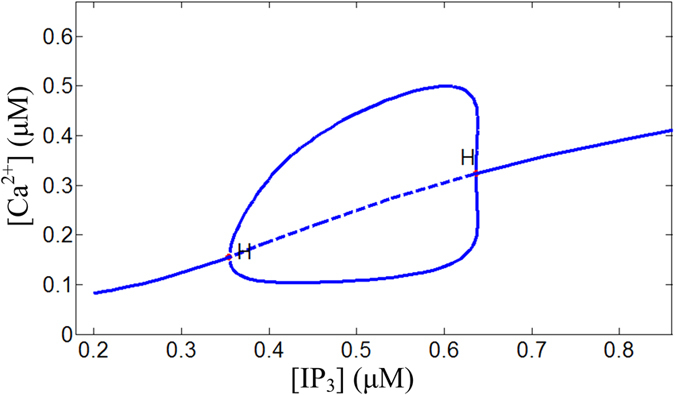
Bifurcation diagram of astrocytic [Ca^2+^] versus the parameter [IP_3_].

The astrocyte, as a source of AG, can be activated by the increase of [IP_3_] concentration. Figure 4 shows the bifurcation diagram of astrocytic caclium oscillation of the Li-Rinzel model^56^. It can be seen that a periodical repetitive calcium oscillation coexists with a steady-state limit cycle for 0.345 μM < [IP3] < 0.664 μM. For [IP3] > 0.664 μM or [IP3] < 0.345 μM, the repetitive calcium oscillation is transformed into resting state.

**Figure 5:**
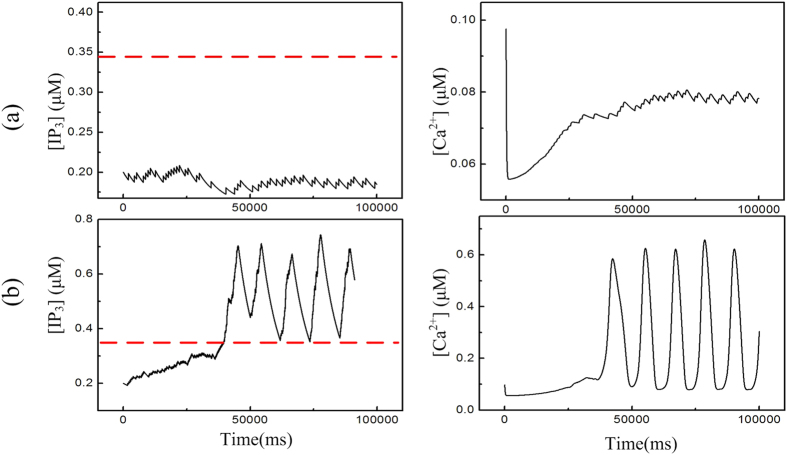
Phase variables of astrocyte system ([IP_3_], [Ca^2+^]) responses to the increase of [AGlu]* with *τ*_aglu_ = 10 s. (**a**) [AGlu]* = 0.0 μM; (**b**) [AGlu]* = 0.5 μM.

In Fig. 5, we show the time course of [IP_3_] and the corresponding calcium oscillation for [AGlu]* = 0.0 μM and 0.5 μM respectively. When [AGlu]* = 0 μM in Fig. 5(a), the [IP_3_] keeps lower than 0.345 μM along the entire period of time. Therefore, [Ca^2+^] stays in a resting state with small amplitude oscillation. However, when [AGlu]* = 0.5 μM in Fig. 5(b), [IP_3_] builds up over the threshold line and calcium oscillations are induced. As a consequence, the high-AG-modulated seizure-like discharge is induced, as shown in Fig. 3. This suggests that the excitable calcium oscillation is a fundamental element for the induction of an epileptic seizure, which has already been proved by experimental results^65^,^66^,^67^,^68^.

**Figure 6:**
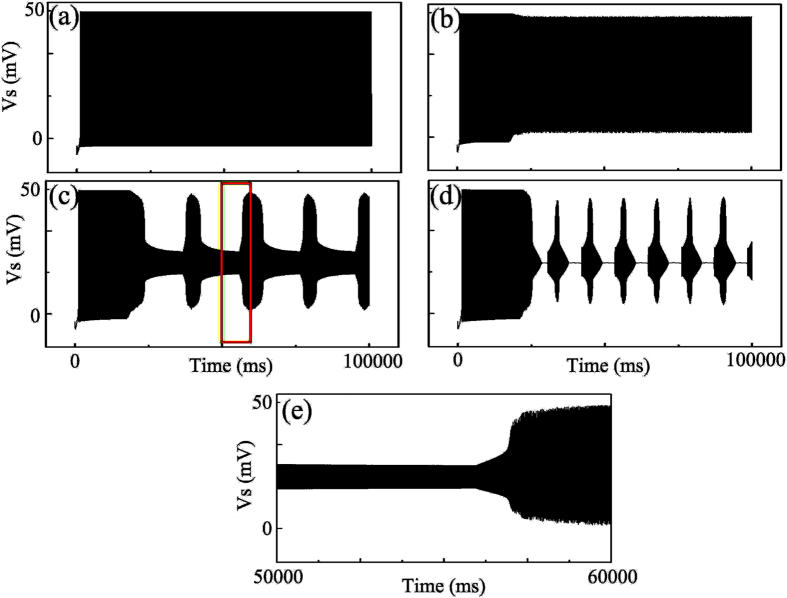
Epileptic discharges induced by increase of time constant *τ*_aglu_ with [AGlu]* = 1.0 μM. Neuronal discharges from top to the bottom with time constant (**a**) *τ*_aglu_ = 2.0 s; (**b**) *τ*_aglu_ = 4.0 s; (**c**) *τ*_aglu_ = 6.0 s; (**d**) *τ*_aglu_ = 8.0 s respectively; (**e**) the partial neuronal discharge zoomed from red square in (**c**).

Figure 6 shows the neuronal discharges for *τ*_aglu_ = 2 s, *τ*_aglu_ = 4 s, *τ*_aglu_ = 6 s and *τ*_aglu_ = 8 s when [AGlu]* = 1.0 μM. We can observe that the regular spiking is transformed into seizure-like discharge when the time constant increases from 2.0 s to 8.0 s, as seen in (d). Besides, Fig. 6(c) shows a “transition state” discharges: the neuronal discharge is a mixture of high-amplitude and low-amplitude phases. The “transition state” discharge has been the prelude of the seizure-like discharges. From the enlargement presented figure in Fig. 6(e), we can see that the low-amplitude phase observed in the “transition state” discharge shows a much higher firing frequency than that of the high-amplitude phase. By comparing the “transition state” discharge with the seizure-like discharges in (d), we concluded that the low-amplitude phase in the “transition state” discharge may directly evolve into the DB state when *τ*_aglu_ increases. In fact, a long time constant of AG clearance process represents another abnormal state of the AG clearance process. Therefore, a prolonged *τ*_aglu_ could also predict the epileptic phenomena, which is shown in the results presented above.

**Figure 7:**
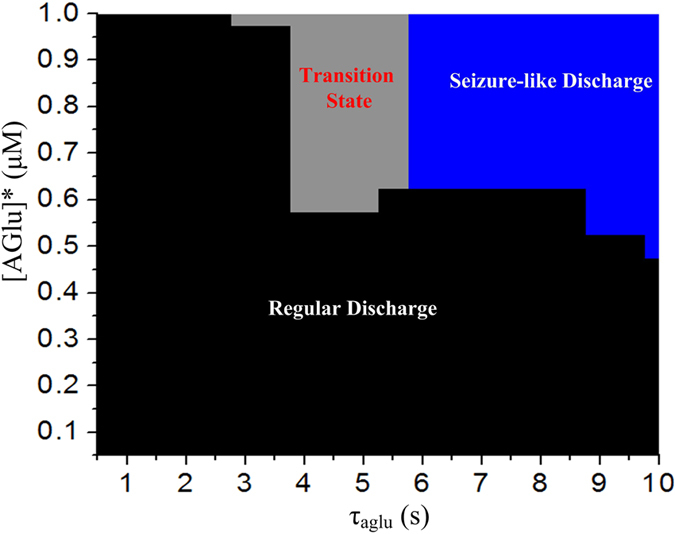
The discharge- state- distribution of the soma in the parameter space of *τ*_aglu_ and [AGlu]*. The black part denotes the regular discharge, the grey part represents the transition state, and the blue part denotes the seizure-like discharge.

In fact, the discharge pattern of soma alternates between seizure-like discharges, transition state activity and regular firing. Figure 7 shows the discharge-pattern distribution of soma in the 2- parameter space, [AGlu]* and *τ*_aglu_. We can see that the seizure-like discharges are present when [AGlu]* or *τ*_aglu_ is high. But if [AGlu]* ≤ 0.5 μM (or *τ*_aglu_ ≤ 5.5 s), increasing the other parameter (*τ*_aglu_ or [AGlu]*) fails to induce the seizure-like discharges. Indeed, the neuronal system often shows some robustness to disordered states (e.g., epilepsy), through mechanisms such as autapse^10^,^11^,^12^,^13^,^14^,^15^,^16^,^17^. Therefore, the epileptic generation thresholds *in vivo* may be higher than the thresholds predicted in our results.

## Conclusions

Prior experimental studies have documented the significant role of astrocytes and their releasing gliotransmitter in epileptic seizure of neurons. In this study, we introduced a model describing the dynamic changes of astrocytic glutamate and we mainly discussed abnormal degradation of extracellular astrocytic glutamate dynamics and its underlying seizure-like discharges in soma. We found that when the equilibrium concentration of the astrocytic glutamate is elevated or when the degradation time constant is lengthened, a seizure-like discharge pattern can be observed in soma action potentials. Besides, the transition from a seizure-like discharge pattern to a regular discharge pattern cannot be induced if one of these two parameters is small enough, as shown in this two-parameter space for the firing-state distribution (Fig. 7).

In addition, by comparing the astrocytic glutamate with the corresponding action potential of soma during the same time window, we found that when the equilibrium concentration is high, the astrocytic glutamate shows an oscillation with a high-level concentration. The peak of astrocytic glutamate concurs with a short period of hyper-resting discharge of the neuron, i.e., a “depolarization block”. Moreover, a lower astrocytic glutamate concentration phase contributes to the high-rate mixed-mode oscillation discharges, i.e., a “refractory status epilepticus” (RSE)-like discharge.

Moreover, by analyzing the energy cost of somatic firing, we found that the somatic firing pattern transition from regular discharges to seizure-like discharges coexists with an “energy explosion”, that is, the seizure-like discharges consume much more energy than regular discharges.

Our modeling work predicts the seizure-like discharge pattern when the astrocytic glutamate degradation process is abnormal. This suggests a pathway for epilepsy when the actual activity of astrocytic transporters is suppressed. Finally, our results provide a better understanding of the role of astrocytes in the induction of epileptic seizure, which could also offer valuable references for experimental and clinical antiepileptic process.

However, due to the lack of experimental data describing the oscillation of astrocytic glutamate concentration, we only developed a linear model describing the dynamics of astrocytic glutamate concentration. Given the complex evolution process of astrocytic glutamate concentration, the non-linearity and/or stochastic processes should also be taken into account in future studies.

## Additional Information

**How to cite this article**: Li, J. *et al*. Dynamic transition of neuronal firing induced by abnormal astrocytic glutamate oscillation. *Sci. Rep*. **6**, 32343; doi: 10.1038/srep32343 (2016).
